# Glycans – the third revolution in evolution

**DOI:** 10.3389/fgene.2014.00145

**Published:** 2014-05-23

**Authors:** Gordan Lauc, Jasminka Krištić, Vlatka Zoldoš

**Affiliations:** ^1^Department of Biochemistry and Molecular Biology, Faculty of Pharmacy and Biochemistry, University of ZagrebZagreb, Croatia; ^2^Genos GlycoscienceZagreb, Croatia; ^3^Department of Molecular Biology, Faculty of Science, University of ZagrebZagreb, Croatia

**Keywords:** glycosylation, evolution, genetics, epigenetics

## Abstract

The development and maintenance of a complex organism composed of trillions of cells is an extremely complex task. At the molecular level every process requires a specific molecular structures to perform it, thus it is difficult to imagine how less than tenfold increase in the number of genes between simple bacteria and higher eukaryotes enabled this quantum leap in complexity. In this perspective article we present the hypothesis that the invention of glycans was the third revolution in evolution (the appearance of nucleic acids and proteins being the first two), which enabled the creation of novel molecular entities that do not require a direct genetic template. Contrary to proteins and nucleic acids, which are made from a direct DNA template, glycans are product of a complex biosynthetic pathway affected by hundreds of genetic and environmental factors. Therefore glycans enable adaptive response to environmental changes and, unlike other epiproteomic modifications, which act as off/on switches, glycosylation significantly contributes to protein structure and enables novel functions. The importance of glycosylation is evident from the fact that nearly all proteins invented after the appearance of multicellular life are composed of both polypeptide and glycan parts.

## GLYCANS ARE ONE OF FOUR MAJOR GROUPS OF MACROMOLECULES

Carbohydrates are one of four major groups of biologically important macromolecules that can be found in all forms of life. They have many biochemical, structural, and functional features that could provide a number of evolutionary benefits or even stimulate or enhance some evolutionary events. During evolution, carbohydrates served as a source of food and energy, provided protection against UV radiation and oxygen free radicals and participated in molecular structure of complex organisms. With time, simple carbohydrates became more complex through the process of polymerization and evolved novel functions. According to the one origin of life theory, called glyco-world, carbohydrates are thought to be the original molecules of life, which provided molecular basis for the evolution of all living things (Stern and Jedrzejas, 2008). Ribose and deoxyribose are integral parts of RNA and DNA molecules and cellulose (glucose polymer) is the most abundant molecule on the planet. There is also evidence for catalytic properties of some carbohydrates (Del Valle, 2004) which further support theory about the capacity of glycans to enable evolution of life.

Carbohydrates are essential for all forms of life, but the largest variety of their functions is now found in higher eukaryotes. The majority of eukaryotic proteins are modified by cotranslational and posttranslational attachment of complex oligosaccharides (glycans) to generate the most complex epiproteomic modification – protein glycosylation. Very large number of different glycans can be made by varying number, order and type of monosaccharide units. The most abundant monosaccharides that can be found in animal glycan are: fucose (Fuc), galactose (Gal), glucose (Glu), mannose (Man), *N*-acetylgalactosamine (GalNAc), *N*-acetylglucosamine (GlcNAc), sialic acid (Sia) and xylose (Xyl). There are two main ways for protein modification with glycans: *O*-glycosylation and *N*-glycosylation. In *O*-glycosylation, the glycan is bound to the oxygen (O) atom of serine or threonine amino acid in the protein. Another type of protein glycosylation is *N*-glycosylation, where glycan is bound to the nitrogen (N) atom of asparagine amino acid in the protein.

Surfaces of all eukaryotic cells are covered with a thick layer of complex glycans attached to proteins or lipids. Many cells in our organism can function without the nuclei, but there is no known living cell that can function without glycans on their surface. Anything approaching the cell, being it a protein, another cell, or a microorganism, has to interact with the cellular glycan coat (Gagneux and Varki, 1999; Varki and Lowe, 2009; Varki, 2011). This appears to be a universal rule since even in sponges, which are the simplest multicellular organisms formed by more or less independent cells, the recognition between cells is based on glycans (Misevic and Burger, 1993). One of the critical steps in the evolution of multicellularity was formation of extracellular matrix (ECM; Sachs, 2008; Hynes, 2012). Multicellular life evolved independently multiple times during evolution and there are two main theories how the initial multicellular group of cells was made. The first theory says that individual cell came together to create symbiotic colonies, and another theory is that cells stayed together after cell division (Sachs, 2008). Appearance of extracellular matrix enabled this initial group of cells to start function as a coordinated unit. Extracellular matrix has huge importance for multicellular organisms (Hynes, 2009). It has role in cell signaling, communication between cells, cell adhesion and in transmitting signal from the environment, and also provides structural support for cells, tissues and organs. Extracellular matrix plays essential role in numerous fundamental processes such as differentiation, proliferation, survival and migration of cells. The main components of ECM are glycoproteins and proteoglycans and the same molecules are responsible for functional properties of ECM (Hynes and Naba, 2012). Extracellular matrix evolved in parallel with first multicellular organisms (Hynes, 2012), therefore, glycans of the early ECM probably participated in evolution of multicellular organisms by enabling communication between cells and thus provided signals for cooperation and differentiation.

In addition to ECM, glycans also provide other properties and functions important for development of multicellular organisms. For example, in some fungal species the sugar *N*-acetylglucosamine (GlcNAc) acts as a signal that stimulates unicellular fungi to start to grow in a multicellular filamentous form (Simonetti et al., 1974; Naseem et al., 2012). The same sugar modifies proteins in multicellular organisms and some of these modifications represent an important mechanism for the regulation of signaling, for example signaling through Notch protein (Rana and Haltiwanger, 2011).

## GLYCANS PERFORM NUMEROUS FUNCTIONS IN COMPLEX ORGANISMS

In complex organisms like humans, glycans play an important role in virtually all processes that involve more than one cell (Walt et al., 2012). Nearly all membrane and secreted proteins are modified by covalent addition of glycans with very high site occupancy (Zielinska et al., 2010). Absence of glycosylation is embryonically lethal (Marek et al., 1999) and mutations which obstruct proper glycosylation cause debilitating diseases (Freeze, 2006). This is not surprising since glycan parts of (glyco) proteins are integral elements of the final molecular structure and together with amino acids in the polypeptide backbone they form a single molecular entity that performs biological functions. Contrary to other posttranslational modifications that generally function as on/off switches, glycosylation generates large complex structures with more profound functions. The role of glycans in biological process should not be ignored since large part of the picture is missing when proteins are being studied without its glycans, or with wrong glycans attached during production of recombinant proteins in non-native organisms, cell types or cellular environment. Unfortunately this is often being done and consequently we are missing essential biological information for many physiological processes.

Two large obstacles in the study of glycans are their non-linear complex chemical structure and the absence of a direct genetic template. Contrary to polypeptides, which are a direct translation of the corresponding gene, glycans are encoded in a complex dynamic network comprising hundreds of genes (Nairn et al., 2008; Lauc et al., 2010). In addition to variation in nucleotide sequence of all genes in this complex biosynthetic pathway, numerous past and present environmental factors also affect the final outcome (Knežević et al., 2010; Lauc and Zoldoš, 2010). However, despite the absence of a direct genetic template, heritability of glycome composition was reported to be high (Knežević et al., 2009; Pucic et al., 2011), even over 80% for some of the glycans in the IgG glycome (Menni et al., 2013).

**FIGURE 1:**
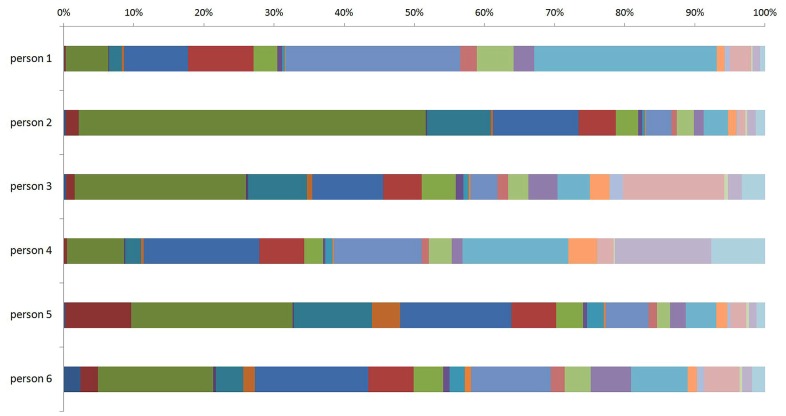
**Inter-individual difference in composition of the glycome attached to immunoglobulin G (IgG) between six individuals.** The term glycome refers to the sum of all glycans that are attached to IgG molecules within an individual. The different (arbitrary) colors represent different *N*-glycan structures that can be attached to IgG molecules within an individual.

**FIGURE 2:**
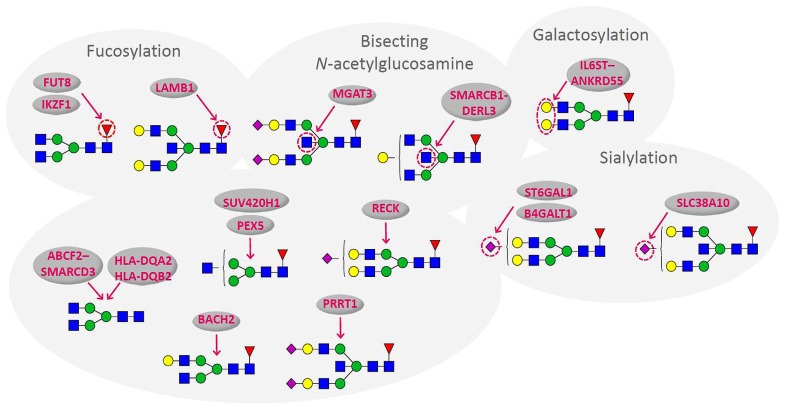
**Genes that associate with variations in the composition of the IgG glycome.** Arrows indicate the part of glycan structure with which particular gene associates (Lauc et al., 2013). Blue square – *N*-Acetylglucosamine; green circle – mannose; yellow circle – galactose; red triangle – fucose; purple diamond – sialic acid.

Recent population studies of the human plasma glycome revealed very large inter-individual differences in glycome composition (Knežević et al., 2009). Interestingly, the variability in composition of the glycome attached to immunoglobulin G (IgG; **Figure 1**) was nearly three times larger than the variability of the total plasma glycome (Pucic et al., 2011), indicating that the variation in concentration of different plasma proteins is blurring the intricate regulation of glycosylation of an individual protein. Genome wide association studies (GWAS) performed in the same populations revealed the similar situation. GWAS of the plasma glycome in 2705 individuals identified three genetic loci that associate with variation in glycosylation (Lauc et al., 2010), while GWAS of the IgG glycome in 2247 individuals identified 16 genetic loci that associate with variations in the composition of the IgG glycome (Lauc et al., 2013; **Figure 2**). This indicates that at least three to four times more than currently estimated 750 genes (Nairn et al., 2008), or at least 10% of the genome participates in glycosylation.

## GLYCANS PROVIDE HIGHER EUKARYOTES WITH UNIQUE ADVANTAGES

Glycosylation, as the most complex epiproteomic modification, gives higher organisms some unique advantages. For example, IgG is one of the most important weapons in our “arsenal,” which enables us to successfully fight with microorganisms, despite their high mutation and reproduction rates. Evolution has invented elaborate genetic mechanisms to create variability in the Fab regions of immunoglobulins, but even more elaborate physiological mechanism of the immune system are activated downstream from the antigen-antibody recognition event (Marchalonis et al., 2002). Since genes for the variable regions of immunoglobulins are by large defined before the first encounter with their target, polypeptide part of the antibody cannot be tuned to the type of antigen it will recognize. However, different invaders (toxins, viruses, bacteria, fungi, parasites) require activation of different effector mechanisms, and this is where protein glycosylation seems to be essential (Nimmerjahn and Ravetch, 2008; Mihai and Nimmerjahn, 2012).

Each heavy chain of IgG carries a single covalently attached bi-antennary *N*-glycan at the highly conserved asparagine 297 residue. The attached glycans are essential structural components of the Fc region and minute changes in glycan composition can significantly change the conformation of the Fc region with dramatic consequences for IgG effector functions. For example, the addition of a fucose residue to the first *N*-acetylglucosamine in the core of the glycan (core-fucose) modifies the conformation of the Fc region in a way to dramatically reduce its ability to bind to FcγRIIIa (Iida et al., 2006; Masuda et al., 2007). FcγRIIIa (CD16) is an activating Fc receptor expressed primarily on natural killer (NK) cells. Through binding to FcγRIIIa IgG initiates antibody-dependent cellular cytotoxicity (ADCC) resulting in the destruction of target cells. On average over 95% of circulating IgGs are core-fucosylated (Pucic et al., 2011) and therefore contain a “safety switch” which prevents them from eliciting potentially destructive ADCC (Scanlan et al., 2008). The small fraction of IgG molecules which lack core-fucose are over 100 times more effective in initiating ADCC through FcγRIIIa binding and this also seems to be the primary mode of function of therapeutic anti-cancer monoclonal antibodies (Shinkawa et al., 2003; Preithner et al., 2006). Improper regulation of this process could lead to either autoimmunity (too much ADCC) or cancer (inefficient ADCC).

Another structural alteration of the IgG glycan, the addition of sialic acid to the ends of glycans changes the function of IgG and converts it from being pro-inflammatory into an anti-inflammatory agent (Kaneko et al., 2006). Sialylation of IgG was found to be essential for the function of intravenous immunoglobulin (IVIG): its anti-inflammatory activity is contained within the effector Fc portion, as Fc fragments alone were found to be sufficient to suppress inflammation (Debre et al., 1993). It appears that Fc with sialylated glycans suppresses inflammation through a novel T_H_2 pathway, which provides an intrinsic mechanism for maintaining immune homeostasis (Anthony et al., 2011). In addition to sialic acids, galactosylation was also shown to be important for the anti-inflammatory activity of IgG (Karsten et al., 2012). Recently reported study by Ackerman et al. (2013) represents just one of the examples how variations in IgG glycosylation can provide new adaptive mechanism that allows fight against pathogens and assure survival. In this study it was shown that people called elite controllers, who are able to control HIV infection and therefore do not get sick, have glycosylation of HIV-specific antibody that promotes strong pro-inflammatory response against the virus (Ackerman et al., 2013). By delegating these tasks to glycans, evolution has created a novel mechanism that enables creation of new structures (which determine antibody function) without the need to alter genetic information. Other glycoproteins were not studied in so much detail as IgG has been, but numerous other examples confirm essential roles of alternative protein glycosylation in many biological processes (Gornik et al., 2012). Among others, binding of glycosylated ligands to selectins is the basis of lymphocyte homing (Walcheck et al., 1996; Sperandio et al., 2009) and alternative glycosylation of Notch is essential for embryonic and adult development (Stanley, 2007). Notch protein is the main actor in Notch signaling pathway that play role in proper development of multicellular organisms. Notch is a transmembrane receptor composed of extracellular, transmembrane and intracellular domains. Upon ligand binding intracellular domain is cleaved and recruited into the nucleus to regulate expression of target genes (Artavanis-Tsakonas et al., 1999). The glycosylation of extracellular domain of Notch protein has impact on ligand recognition and activation of Notch signaling. Four different types of glycans have been reported to be present on Notch: *N*-glycans, *O*-fucose glycans, *O*-glucose glycans, and *O*-GlcNAc glycans, but just two of them are known to significantly affect and modulate Notch activity: *O*-fucose glycans and *O*-glucose glycans. The *O*-fucosylation of Notch extracellular domain is found to be important in ligand recognition and binding (Stanley, 2007; Rana and Haltiwanger, 2011). Further extension of *O*-fucose by the addition of *N*-acetylglucosamine, in a reaction catalyzed by glycosyltransferase called Fringe, also modulates Notch activity. Through the action of Fringe, Notch signaling pathway can be induced or inhibited depending on the type of ligand that interacts with Notch receptor (Hicks et al., 2000). With respect to the role of Notch signaling in differentiation of wide range of cell types it is evident that alterations in pattern of glycosylation could provide mechanism by which different signals could be received through the same or homologous signaling proteins. We can expect new insights into the importance of glycosylation on Notch signaling, starting with recently reported study which has pointed out that Notch protein can also carry O-linked GalNAc glycans with the role in formation of body laterality (Boskovski et al., 2013). Another example of role of glycosylationin signaling during development of multicellular organisms is *O*-fucosylation of Crypto protein. Crypto protein is one of the proteins involved in Nodal signaling pathway and proper glycosylation of this protein is important for its activity and binding properties (Schier and Shen, 2000; Yan et al., 2002).

## GLYCANS ENABLE DYNAMIC EPIGENETIC ADAPTATION

It is generally assumed that the appearance of self-replicating nucleic acids (the first revolution in evolution) provided the basis for the development of early life. Nucleic acids then recruited amino acids to create proteins, which are still the main effectors of life at the cellular level (the second revolution). However, the integration of different cells into a complex multicellular organism required an additional layer of complexity. Here we propose that the invention of protein glycosylation (the third revolution) through its inherent ability to create novel structures without the need to alter genetic information enabled the development of multicellular life in its present complexity.

The biggest evolutionary advantage that glycans confer to higher eukaryotes is the ability to create new structures without introducing changes into the precious genetic heritage (Lauc and Zoldoš, 2010; Lauc et al., 2013). In principle all posttranslational modifications enable this to some extent, but most of them function as simple on/off molecular switches, while glycans represent significant structural components contributing with up to 50% in mass (Pierce and Parsons, 1981) and even much more to the molecular volume of many proteins (Bush et al., 1999). The fact that so large parts of the molecule are not hardwired in the genome provides a rapid and extensive epiproteomic adaptation mechanism. One example of role of glycosylation in the process of adaptation is found to be important for function of mammalian sperm cell and for the reproduction process itself. Mammalian sperm cells are masked with sialylated sugars in order to prevent recognition as foreign cells in the female reproductive system. After successful adaptation of sperm cell to the new environment, the removal of sialic acid residues from sperm surface glycans is the necessary step in the process of sperm cell maturation and the establishment of interaction between sperm and egg cells (Ma et al., 2012). Another interesting example how glycosylation of proteins can ensure adaptation and survival comes from the kingdom of archaebacteria (Guan et al., 2012).

Epigenetic regulation of gene expression has been reported to be important for protein glycosylation (Saldova et al., 2011; Horvat et al., 2012, 2013; Zoldoš et al., 2012) and this could explain the observed temporal stability of the glycome (Gornik et al., 2009). Comparative studies of the glycome in different organisms are rare, but they indicate higher rates of divergence in glycans than in proteins or DNA (Royle et al., 2008; Zielinska et al., 2012; Adamczyk et al., 2014). Interactions established through glycans are not restricted just to cell- cell interactions and communication that could have played significant role in the evolution of multicellular life forms. Glycans also play significant role in the interaction between different organisms, including host-pathogen interactions or interactions between symbionts. Effect of glycosylation on the composition of the human intestinal microbiota has been well examined (Koropatkin et al., 2012). Intestinal symbiotic bacteria are very important to humans as they help in food digestion, produce some vitamins and provide protection against pathogenic bacteria. In return, symbiotic bacteria use host glycan molecules as receptors for colonization of intestine and, also, both host and dietary glycans serve as energy source for symbiotic bacteria. It is reported that individuals who don’t secrete blood group glycans into the intestinal mucosa have reduced number and diversity of probiotic bacteria in the intestine (Wacklin et al., 2011). Except for food, symbiotic bacteria also use sugars that are highly abundant in intestine for glycosylation of their surface in order to escape the human immune system (Coyne et al., 2005). Furthermore, digestion of sugars by symbiotic bacteria enables activation of signaling system that control pathogenicity of some non-symbiotic bacteria (Pacheco et al., 2012). Based on these facts, it can be safely assumed that glycans play important role in evolution of symbiotic relationship between humans and intestinal bacteria.

In some biological systems, like for example AB0 blood groups, glycans act as simple molecular switches that introduce inter-individual variability of cellular surfaces. In other systems, like immunoglobulin glycosylation, they enable new physiological functions, which could not be performed without this complex posttranslational tool. Glycosylation is particularly complex in human brain, but currently available technologies do not allow detailed study of this highly intricate system. Since all eukaryotic cells are heavily glycosylated (at significant metabolic cost) and elaborate mechanisms that regulate glycosylation are being discovered, we propose that the invention of glycosylation was the third large revolution in evolution, which enabled the development of complex multicellular organisms.

## Conflict of Interest Statement

The authors declare that the research was conducted in the absence of any commercial or financial relationships that could be construed as a potential conflict of interest.
